# Novel Mutation in the Calcium-Sensing Receptor Gene Associated With Familial Hypocalciuric Hypercalcemia

**DOI:** 10.7759/cureus.66498

**Published:** 2024-08-09

**Authors:** Hadeel A Al Kayed, Saif U Islam, Olayemi J Akanmode, Lynda A Ezike, Lubna Mirza

**Affiliations:** 1 Medicine and Surgery, The University of Jordan, Amman, JOR; 2 Clinical Sciences, Avalon University School of Medicine, Willemstad, CUW; 3 General Medicine, King’s College Hospital NHS Foundation Trust, London, GBR; 4 General Medicine, Garki Hospital, Garki, NGA; 5 General Medicine, Kursk State Medical University, Kursk, RUS; 6 Endocrinology, Norman Regional Hospital, Norman, USA

**Keywords:** genetic testing, hypocalciuria, hypercalcemia, casr gene, familial hypocalciuric hypercalcemia

## Abstract

We present a case of a 75-year-old woman with persistent hypercalcemia (serum calcium 10.7 mg/dL, ionized calcium 1.37 mmol/L), elevated parathyroid hormone levels (86.6 pg/mL), and significantly low 24-hour urinary calcium excretion (<11 mg/24 hours). Genetic testing identified a novel heterozygous variant in the calcium-sensing receptor (CaSR) gene, c.3166G>C (p. Val1056Leu). The patient's biochemical profile and the identification of the CaSR variant support the diagnosis of familial hypocalciuric hypercalcemia (FHH). The novel c.3166G>C (p.Val1056Leu) variant has not been previously reported in FHH or other CaSR-associated conditions. Its presence in this patient suggests a potential role in the clinical manifestation of FHH. However, it is currently classified as a variant of undetermined significance (VUD) in the ClinVar database, necessitating further research on the clinical relevance of this variant in FHH.

This case highlights the significance of genetic testing in diagnosing FHH and the potential clinical impact of discovering novel CaSR gene mutations. Further research on the genetics associated with FHH is necessary to better understand the condition, detect it early, and manage it effectively, thereby improving patient care and outcomes.

## Introduction

Familial hypocalciuric hypercalcemia (FHH) is a rare, benign genetic disorder that typically presents with mild to moderate hypercalcemia, low urinary calcium excretion, and normal to slightly elevated levels of parathyroid hormone (PTH). This condition is usually asymptomatic and is often discovered incidentally during routine blood tests. Therefore, although its prevalence has been estimated to be in the range of one in 78,000, the true prevalence is likely to be higher. FHH can be diagnosed at any age, with the severe forms presenting in infancy [1].

FHH is typically inherited in an autosomal dominant manner, meaning that inheriting a single copy of the mutated gene from one parent is sufficient to cause the disorder. This inheritance pattern is characterized by variable expressivity, where the severity of symptoms can vary among individuals, and high penetrance, meaning most individuals who inherit the mutation will exhibit some form of the condition [2].

The management of FHH primarily involves regular monitoring, given the typically benign nature of the condition, which usually does not necessitate treatment. Patients are advised to avoid unnecessary surgical interventions. Genetic counseling is recommended for affected families to provide education and reassurance about the benign nature of FHH. Calcimimetics, allosteric activators of the calcium-sensing receptor (CaSR), represent a potential therapeutic approach for conditions involving hypoactive CaSR, such as FHH [1,3].

FHH is often caused by a heterozygous loss-of-function mutation in the CaSR gene. This gene encodes a G-protein coupled receptor known as the calcium-sensing receptor (CaSR), which is crucial for maintaining calcium homeostasis by regulating parathyroid hormone (PTH) secretion and calcium excretion in the kidneys [1,3].

CaSRs are primarily located on the parathyroid glands but also in the kidneys and other tissues. Under normal conditions, low calcium levels stimulate CaSRs to increase PTH secretion and enhance calcium reabsorption in the kidneys. Mutations in the CaSR gene reduce the sensitivity of these receptors to calcium, necessitating higher-than-normal serum calcium levels to suppress PTH release. Additionally, in the kidneys, this defect leads to increased tubular calcium and magnesium reabsorption (i.e., reduced excretion), resulting in hypercalcemia, hypocalciuria, and often high-normal levels of serum magnesium [3].

To date, over 300 mutations in the CaSR gene have been associated with FHH [3], with the majority being loss-of-function mutations [1]. These mutations include missense, nonsense, and frameshift mutations, each affecting the receptor's function to varying degrees [2]. In this case report, we describe a patient with FHH presenting a previously unreported heterozygous variant in the CaSR gene: c.3166G>C (p.Val1056Leu). This specific mutation is absent from population databases and has not been documented in the literature in individuals with FHH or other CaSR-related conditions [4].

## Case presentation

A 75-year-old woman was referred to the Endocrine Center at Norman Regional Health System by her primary care physician for evaluation of hypercalcemia and hyperparathyroidism. The patient has a known history of hypothyroidism, for which she has been receiving thyroid hormone replacement therapy for many years. In September 2023, her serum calcium level was found to be elevated at 10.4 mg/dL. A subsequent comprehensive metabolic panel (CMP) conducted in February 2024 confirmed persistent hypercalcemia with a serum calcium level of 10.7 mg/dL and an intact PTH level of 116.5 pg/mL. The patient reported no family history of calcium disorders or Ambien syndrome, but given the typically asymptomatic nature of FHH, this information should be interpreted with caution.

Her past medical history includes hypothyroidism for several years, for which she takes levothyroxine and maintains a normal thyroid stimulating hormone (TSH) range. Additionally, her medical history includes kidney stones, gastroesophageal reflux disease (GERD) with gastrointestinal bleeding, lumbar spine injury, skin cancer on the forehead, anemia, hypertension, morbid obesity, and restless leg syndrome. Her surgical history includes a right total knee arthroplasty, cholecystectomy, two cesarean sections, hysterectomy, back spinal fusion, gastric bypass, tonsillectomy, root canal, and back surgery.

The patient's family history includes hypertension in her father and diabetes mellitus, hypertension, cancer, and stroke in her mother. Additionally, her siblings have seizure disorders.

The patient's current medications include a multivitamin, alpha-lipoic acid, vitamin B12, thiamine, zinc, iron sulfate, omega-3, levothyroxine, hydrochlorothiazide, ondansetron, omeprazole, simethicone, hydrocodone-acetaminophen, senna, and lactulose solution. The patient has allergies to sulfa antibiotics, non-steroidal anti-inflammatory drugs (NSAIDs), tuna fish, and nickel.

On physical examination, the patient was alert and oriented, with no acute distress. No thyroid enlargement or lymphadenopathy was observed. A cardiovascular examination revealed a normal sinus rate and normal heart sounds. Her lungs were clear, and her abdomen was soft, non-tender, and non-distended. There was no edema in her extremities, and her deep tendon reflexes were symmetrical.

Laboratory results indicated hypercalcemia with serum calcium at 8.9 mg/dL and elevated PTH at 115.8 pg/mL. Total vitamin D was low at 26.4 ng/mL. Magnesium and phosphate levels were normal, measuring 2.0 mg/dL and 3.2 mg/dL, respectively. Refer to Table 1 for detailed laboratory results.

**Table 1:** Laboratory test results. eGFR: estimated glomerular filtration rate, BUN: blood urea nitrogen, PTH: parathyroid hormone.

Test	Value	Interpretation
Total calcium	8.9 mg/dL	Normal
Glucose	112 mg/dL	High
Magnesium	2.0 mg/dL	Normal
Phosphorus	3.2 mg/dL	Normal
Potassium	4.6 mmol/L	Normal
Sodium	138 mmol/L	Normal
PTH related protein	0.6 pmol/L	Normal
PTH	115.8 pg/mL	High
Total vitamin D	26.4 ng/mL	Low
1,25-dihydroxyvitamin D	48 pg/mL	Normal
BUN	24 mg/dL	High
Creatinine	0.99 mg/dL	Normal
BUN/creatinine ratio	24	High
eGFR	58 mL/min	Low

Subsequent testing revealed persistently elevated serum calcium at 8.9 mg/dL and PTH at 115.8 pg/mL. A 24-hour urinary calcium excretion showed a calcium level of 11 mg/dL and a urinary creatinine level of 1075 mg/dL, with a calcium/creatinine ratio of 0.0102. The total urine volume over 24 hours was 1075 mL. The combination of elevated calcium levels, normal to elevated PTH, and hypocalciuria prompted further genetic testing.

We utilized Invitae, a leading genetic testing company, to perform a multi-gene panel sequencing using next-generation sequencing technology. This approach allows for the simultaneous sequencing of multiple DNA fragments, ensuring high-quality and accurate results [5]. The testing confirmed the presence of a heterozygous c.3166G>C (p.Val1056Leu) variant in the CaSR gene.

This variant has not been previously reported in individuals with FHH and is not present in population databases [4]. However, considering that mutations in the CaSR gene are strongly linked to FHH, combined with the distinctive biochemical findings observed in this patient, this strongly suggests a diagnosis of FHH.

The patient was counseled on the benign nature of FHH and the avoidance of unnecessary surgical interventions. To address the patient's vitamin D deficiency, high-dose vitamin D supplementation was prescribed, including 600 mg of calcium twice daily and 2000 IU of vitamin D3 daily. The patient was also encouraged to increase her dietary intake of calcium-rich foods. Lifestyle modifications to prevent falls were advised, and follow-up on bone density assessment was planned if clinically indicated.

## Discussion

FHH is a genetic disorder frequently caused by inactivating mutations in the CaSR gene. The CaSR gene is crucial for calcium homeostasis, primarily by regulating PTH secretion in response to extracellular calcium levels. Mutations in this gene raise the set point for calcium homeostasis, meaning higher-than-normal serum calcium levels are required to suppress PTH release, resulting in mild to moderate hypercalcemia with normal to high PTH levels. CaSR mutations also increase calcium reabsorption in the kidneys, resulting in decreased urinary calcium levels (hypocalciuria) [1,3].

**Figure 1:**
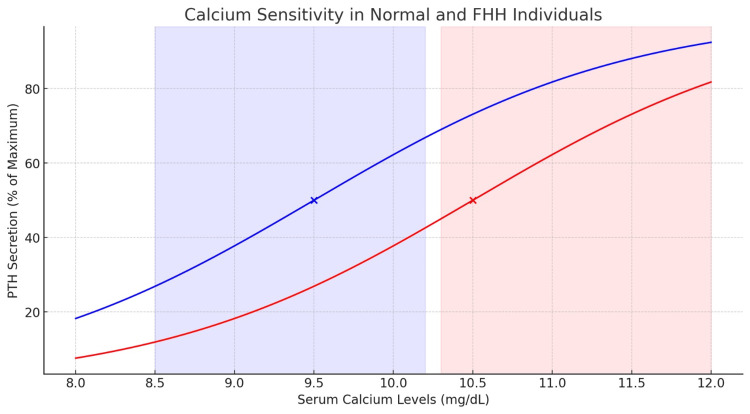
Calcium sensitivity in normal and FHH individuals. The above graph illustrates the relationship between serum calcium concentration and PTH secretion in normal individuals (blue curve, left) and individuals with FHH (red curve, right). The x-axis represents serum calcium levels, while the y-axis represents PTH secretion as a percentage of maximum. The intersection points at %50 PTH secretion are indicated by dots, highlighting the differences in calcium sensitivity. The FHH curve is shifted to the right, indicating a higher calcium set point compared to normal individuals. The shaded areas represent the typical serum calcium ranges for normal (blue) and FHH (red) individuals [3,6]. Figure drawn by Dr. Islam. FHH: familial hypocalciuric hypercalcemia, PTH: parathyroid hormone.

As illustrated in Figure 1, the relationship between serum calcium concentration and PTH secretion in individuals with FHH is shifted compared to normal individuals. The curve representing FHH individuals is shifted to the right, indicating a higher calcium set point due to the decreased sensitivity of the calcium-sensing receptor. This shift explains why individuals with FHH maintain higher serum calcium levels to achieve the same level of PTH secretion as normal individuals.

Differentiating FHH from primary hyperparathyroidism (PHPT) is crucial since both conditions cause hypercalcemia and elevated PTH levels. However, PHPT may require surgical intervention, whereas FHH management focuses on monitoring and genetic counseling, avoiding unnecessary surgeries.

FHH is often diagnosed in asymptomatic hypercalcemic patients with a family history of hypercalcemia, normal serum PTH levels, and very low urinary calcium excretion (<100 mg/24 hours), with a calcium-to-creatinine clearance ratio (Ca/Cr) typically less than 0.01. Genetic testing for CaSR gene mutations is recommended for patients with a Ca/Cr clearance ratio of 0.020 or less. Conversely, PHPT usually presents with elevated PTH levels, higher urinary calcium excretion, and a Ca/Cr clearance ratio greater than 0.02. The calcium infusion test can also help differentiate the two; PHPT shows increased urinary calcium excretion with rising calcium load, unlike FHH. Additionally, serum magnesium levels are typically upper-normal or mildly elevated in FHH but normal or low in PHPT [1].

It is important to consider other conditions that can influence calcium metabolism and potentially cause low urinary calcium excretion. Our patient has several factors that could impact calcium levels: her estimated glomerular filtration rate (eGFR) was 58 mL/min/1.73 m², with elevated blood urea nitrogen (BUN) and a high BUN/creatinine ratio, indicating impaired renal function, which can affect calcium homeostasis and urinary excretion. Additionally, the patient is taking hydrochlorothiazide, a thiazide diuretic known to reduce urinary calcium excretion. The patient also had low vitamin D levels, which can impair calcium absorption and affect overall calcium homeostasis. Furthermore, the patient exhibits malabsorptive symptoms and takes antacids, both of which can influence calcium absorption and excretion. Despite these conditions, which typically do not cause hypercalcemia, the patient's presentation aligns with the characteristic features of FHH. The presence of a novel heterozygous variant in the CaSR gene, c.3166G>C (p.Val1056Leu), further supports the diagnosis. This variant likely decreases the sensitivity of the calcium-sensing receptor, resulting in the observed biochemical profile. While impaired renal function, thiazide use, vitamin D deficiency, and malabsorption can complicate the assessment, they do not fully account for the patient's hypercalcemia and hypocalciuria. Thus, the diagnosis of FHH remains the most consistent with her clinical and genetic findings.

Given the autosomal dominant inheritance pattern of FHH, first-degree relatives of affected individuals have a 50% chance of inheriting the mutation. Genetic testing can help identify carriers and inform clinical management, including regular monitoring of calcium levels and avoiding unnecessary interventions.

Cinacalcet, a calcimimetic agent, is a promising treatment option for symptomatic cases of FHH. Cinacalcet acts as an allosteric modulator of the CaSR, increasing its sensitivity to extracellular calcium and enhancing signal transduction. This can help normalize serum calcium levels in patients with severe hypercalcemia or symptoms related to hypercalcemia [1,3,7].

## Conclusions

The identification of the novel heterozygous variant in the CaSR gene, c.3166G>C (p.Val1056Leu), in a patient presenting with the typical features of FHH, underscores the pathogenic potential of this previously unreported mutation. Despite being documented as a VUD, its location in the CaSR gene, known for its role in FHH, combined with the clinical and biochemical presentation of our patient, strongly supports the diagnosis. Genetic counseling and testing play a crucial role in managing FHH, not only for the affected individuals but also for their families, given the autosomal dominant inheritance pattern of the condition. Early identification of carriers through genetic testing can facilitate proactive clinical management, avoiding unnecessary treatments and enabling regular monitoring of calcium levels. This case contributes to the expanding knowledge of CaSR mutations and emphasizes the need for continued research to understand the genetic diversity and molecular mechanisms underlying FHH.
